# Towards determination of power loss at a rowing blade: Validation of a new method to estimate blade force characteristics

**DOI:** 10.1371/journal.pone.0215674

**Published:** 2019-05-09

**Authors:** Lotte L. Lintmeijer, John P. T. Onneweer, Mathijs J. Hofmijster, Willem A. Wijgergangs, Hans de Koning, Bert Clairbois, Jerry Westerweel, Ernst J. Grift, Mark J. Tummers, A. J. van Soest

**Affiliations:** 1 Amsterdam Movement Science, Vrije Universiteit Amsterdam, Amsterdam, The Netherlands; 2 Laboratory for Aero & Hydrodynamics, Delft University of Technology, Delft, The Netherlands; 3 Center of Applied Research in Sports and Nutrition, Amsterdam University of Applied Sciences, Amsterdam, The Netherlands; Politecnico di Milano, ITALY

## Abstract

To analyze on-water rowing performance, a valid determination of the power loss due to the generation of propulsion is required. This power los can be calculated as the dot product of the net water force vector (${\vec{F}}_{w,o}$) and the time derivative of the position vector of the point at the blade where ${\vec{F}}_{w,o}$ is applied (${\vec{r}}_{PoA/w}$). In this article we presented a method that allows for accurate determination of both parameters using a closed system of three rotational equations of motion for three different locations at the oar. Additionally, the output of the method has been validated. An oar was instrumented with three pairs of strain gauges measuring local strain. Force was applied at different locations of the blade, while the oar was fixed at the oarlock and the end of the handle. Using a force transducer and kinematic registration, the force vector at the blade and the deflection of the oar were measured. These data were considered to be accurate and used to calibrate the measured strain for bending moments, the deflection of the oar and the angle of the blade relative to its unloaded position. Additionally, those data were used to validate the output values of the presented method plus the associated instantaneous power output. Good correspondence was found between the estimated perpendicular blade force and its reference (ICC = .999), while the parallel blade force could not be obtained (ICC = .000). The position of the PoA relative to the blade could be accurately obtained when the perpendicular force was ≥ 5.3 N (ICC = .927). Instantaneous power output values associated with the perpendicular force could be obtained with reasonable accuracy (ICC = .747). These results suggest that the power loss due to the perpendicular water force component can be accurately obtained, while an additional method is required to obtain the power losses due to the parallel force.

## Introduction

For an accurate determination of the average power lost to the generation of propulsion per stroke cycle (${\bar{P}}_{blade}$; see Table 1 for a list of all abbreviations), valid information about the net water force vector at the blade of the oar (${\vec{F}}_{w,o}$) and its associated point of application (PoA) are essential since:

$$\begin{array}{c} {\bar{P}}_{blade}=\frac{1}{T}\int_{t_{0}}^{t_{0}+T}\left({\vec{F}}_{w,o}\cdot {\dot{\vec{r}}}_{PoA/w}\right)\;dt \end{array}$$ (1)

where T is the time duration of a stroke cycle and ${\dot{\vec{r}}}_{PoA/w}$ is the time derivative of the position vector (i.e. the velocity vector) of the point of the blade where ${\vec{F}}_{w,o}$ is applied relative to an earth-bound frame of reference (${\vec{r}}_{PoA/w}$). Determination of ${\vec{F}}_{w,o}$, ${\vec{r}}_{PoA/w}$ and its time derivative is not trivial due to the (1) deflection of the oar and (2) a constantly changing force distribution at the blade resulting in an unknown and time-variant point of application of the water force. In previous studies [1–8], power loss due to the generation of propulsion has been estimated assuming the oar to be rigid and the PoA of the water force vector to be in the middle of the blade. Additionally, the force component parallel to the blade has been typically neglected. These assumptions are not only unrealistic [9, 10], but do also affect calculated values of ${\bar{P}}_{blade}$ significantly [4].

**Table 1:** Abbreviation of parameters.

Abbreviation	Meaning	Units
PoA	The point of application.	
${\vec{F}}_{w,o}$	(simulated) Force vector of the water on the blade.	N
$F_{w,o}^{x}$	The x-component of ${\vec{F}}_{w,o}$ in an earth-bound frame of reference.	N
$F_{w,o}^{y}$	The y-component of ${\vec{F}}_{w,o}$ in an earth-bound frame of reference.	N
$F_{w,o}^{x^{\prime }}$	The x’-component of ${\vec{F}}_{w,o}$ in a blade-bound frame of reference.	N
$F_{w,o}^{y^{\prime }}$	The y’-component of ${\vec{F}}_{w,o}$ in a blade-bound frame of reference.	N
${\vec{r}}_{PoA/w}$	Position vector of the point of the blade where the PoA is located at that time moment relative to the world.	m
$r_{PoA/w}^{x^{\prime }}$	The x’-component of the ${\vec{r}}_{PoA/w}$ relative to the world.	m
$r_{PoA/T}^{x^{\prime }}$	The x’-component of the ${\vec{r}}_{PoA/w}$ relative to the beginning of the blade.	m
${\dot{\vec{r}}}_{PoA/w}$	The time derivative of ${\vec{r}}_{PoA/w}$.	m/s
${\dot{r}}_{PoA/w}^{x^{\prime }}$	The x’-component of the time derivative of ${\vec{r}}_{PoA/w}$.	m/s
${\dot{r}}_{PoA/w}^{y^{\prime }}$	The y’-component of the time derivative of ${\vec{r}}_{PoA/w}$.	m/s
$r_{i}^{x}$	The x-component of the moment-arm from location *i* to *i-1* in an earth-bound frame of reference.	m
$r_{i}^{y}$	The y-component of the moment-arm from location *i* to *i-1* in an earth-bound frame of reference.	m
$r_{i}^{x^{\prime }}$	The x-component of the moment-arm from location *i* to *i-1* in a blade-bound frame of reference.	m
$r_{i}^{y^{\prime }}$	The y-component of the moment-arm from location *i* to *i-1* in a blade-bound frame of reference.	m
$M_{i}^{z}$	The bending moment at location *i* of the oar shaft.	Nm
$\Delta_{oar}^{y}$_*i*_	The y-component of the position of location *i* in the loaded situation relative to its location in the unloaded position.	m
T	The beginning of the blade.	
E	The end of the blade.	
Φ_*b*/*w*_	The angle of the blade relative to its neutral unloaded position.	rad
${\bar{P}}_{blade}$	Power loss due to the generation of propulsion averaged over a stroke cycle.	W
*P*_*defl*_	Power output due to the deflection of the oar shaft.	W
*sg*	*Determined using the presented method with strain gauges*.	
*ref*	*Reference parameters determined using Optotrak and a force transducer*.	

In the first part of this article we present a novel cost-effective method to obtain ${\vec{F}}_{w,o}$ and ${\vec{r}}_{PoA/w}$ that does not rely on the above mentioned assumptions. Additionally, we evaluate whether the method provides an accurate quantification of ${\vec{F}}_{w,o}$ and ${\vec{r}}_{PoA/w}$ in a simulated rowing situation. After showing that both parameters can be determined accurately, an indication of the extent in which ${\bar{P}}_{blade}$ can be accurately determined in on-water rowing is provided.

**Fig 1:**
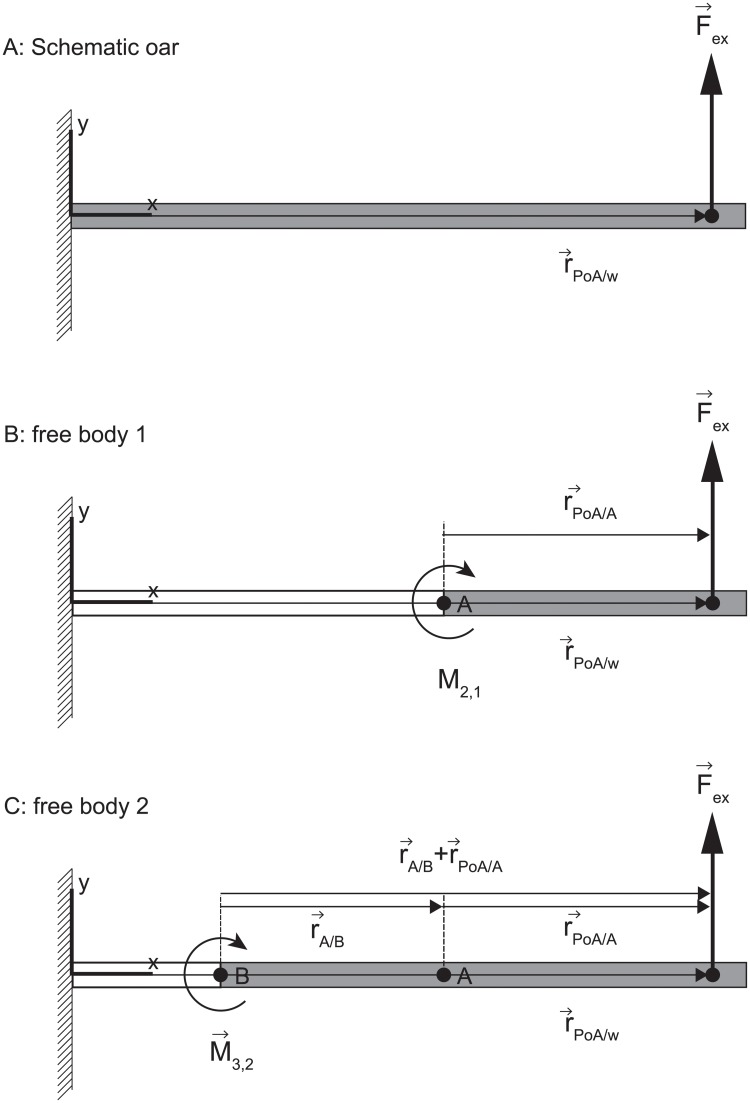
A schematic diagram of an non-deformable oar with a net external force (${\vec{F}}_{ex}$) applied at the free end of the oar and the position of the location at the blade at which the external force is applied at that moment in time (${\vec{r}}_{PoA/w}$) (panel A). In panel B and C the free bodies 1 and 2 are presented.

In the presented method we make use of pairs of light-weight strain gauges that are attached at location *i* at the oar shaft and measure the local bending strain at location *i*. This local strain is a function of the local bending moment and the material properties of the oar shaft [11]. It is straightforward to show that—in concept—this bending moment contains information regarding the net force applied near a free end of the oar and its point of application by analyzing the rotational equation of motion for a free body running from location *i* to the free end of the oar. Consider, as an example, the schematic representation of an oar in an xy-plane in Fig 1. Taking point *A* as the pivot point of free body 1 and measuring the bending moment at point *A*, the following rotational equation of motion for free body 1 is obtained:

$$\begin{array}{c} {\vec{M}}_{2,1}+{\vec{M}}_{F_{ex}}={\vec{M}}_{2,1}+{\vec{r}}_{PoA/A}\times {\vec{F}}_{ex}={\vec{I}}_{1/A}{\ddot{\vec{\phi }}}_{1} \end{array}$$ (2)

where ${\vec{M}}_{2,1}$ and ${\vec{M}}_{F_{ex}}$ are the bending moment vectors measured at location *A* and the unknown moment vector due to a net external force vector, respectively. ${\vec{F}}_{ex}$ is the unknown external force applied at the free end of the oar and ${\vec{r}}_{PoA/A}$ is the unknown point of the oar at which ${\vec{F}}_{ex}$ is applied relative to point A. *I*_1/*A*_ is the inertia of free body 1 and ${\ddot{\vec{\phi }}}_{1}$ the oar angular acceleration of the free body.

As the right hand term of the Eq 2 is relatively small, it can be neglected and a quasi-static approach can be applied, which results in:

$$\begin{array}{c} {\vec{M}}_{2,1}=-{\vec{r}}_{PoA/A}\times {\vec{F}}_{ex} \end{array}$$ (3)

If oar deflection would be neglected, Eq 3 can be further simplified into:

$$\begin{array}{c} M_{2,1}^{z}=-r_{PoA/A}^{x}\cdot F_{ex}^{y} \end{array}$$ (4)

Eq 4 thus provides information on the product of the unknown $r_{PoA/A}^{x}$ and $F_{ex}^{y}$. Note that the moment only has a z-component since the z-component of the force vector is negligible. When a second bending moment at a another location such as at location *B* (see Fig 1C) is measured, a second rotational equation of motion can be formulated with the very same two unknowns:

$$\begin{array}{c} M_{3,2}^{z}=-\left(r_{PoA/A}^{x}+r_{A/B}^{x}\right)F_{ex}^{y} \end{array}$$ (5)

In which $M_{3,2}^{z}$ is the measured internal moment in point *B* and $r_{A/B}^{x}$ the known x-component of the position vector of point *B* relative to point *A*.

Interestingly, Eqs 4 and 5 are independent and—although the relation between the two unknowns is nonlinear -this system of two equations has a unique solution:

$$\begin{array}{cc} F_{ex}^{y} & =\frac{M_{2,1}^{z}-M_{3,2}^{z}}{r_{A/B}^{x}} \end{array}$$ (6a)

$$\begin{array}{cc} r_{PoA/A}^{x} & =-\frac{M_{2,1}^{z}\cdot r_{A/B}^{x}}{M_{2,1}^{z}-M_{3,2}^{z}} \end{array}$$ (6b)

As explained above, Eq 6 is obtained when the oar is assumed to be rigid. When the oar is assumed to be deformable, additional unknown parameters appear. For every extra unknown parameter related to the applied external force vector and the ${\vec{r}}_{PoA/A}$, an extra pair of strain gauges and and extra free body with a related rotational equation of motion with the exact same parameters is prerequisite in order to obtain a system of equations that has a unique solution.

Thus the essence of our method is that we can calculate the values of *n* unknown parameters related to an applied external force vector and the ${\vec{r}}_{PoA/A}$, using a system of *n* independent but nonlinear rotational equations of motions for *n* free bodies with known bending moments. Moreover, any redundant measurement and related rotational equation of motion (*n+1*) will result in an overdetermined system that can be solved using a least square method.

**Fig 2:**
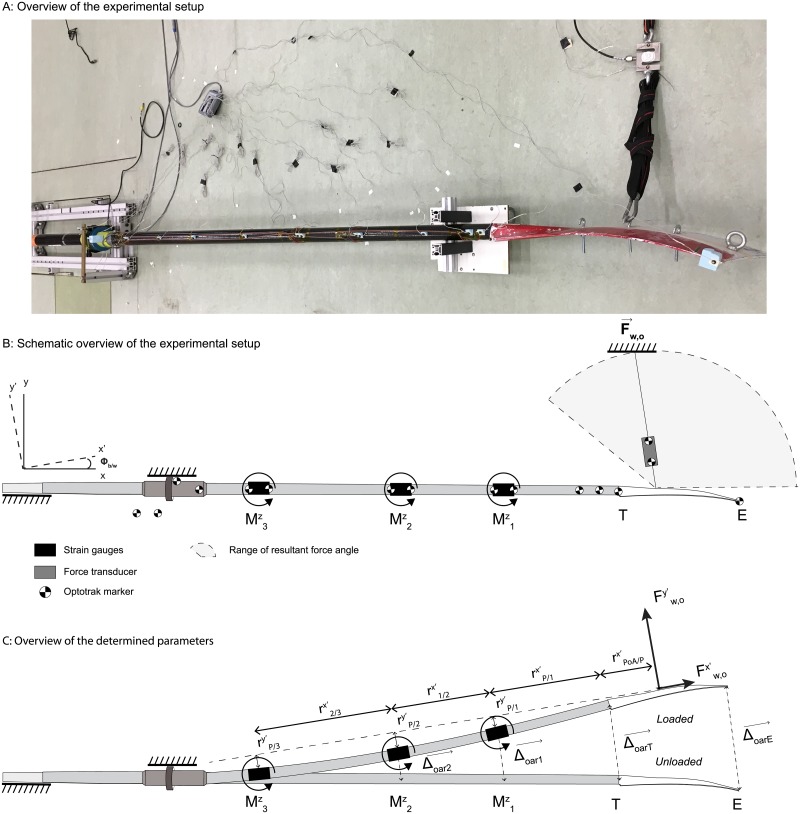
(A) An overview of the experimental setup, (B) the related schematic overview of the experimental setup in a horizontal plane, and (B) a schematic representation of the bended oar relative to its neutral position with the relevant determined parameters. The xy-frame of reference represents an earth-bound frame of reference in which the positive x-axis points towards the blade of the oar in an unloaded position. The x’y’-frame of reference represents the blade-bound frame of reference in which the positive x’-axis points towards the end of the blade.Φ_*b*/*w*_ is the angle of the blade in loaded position relative to the blade in neutral position. $M_{1}^{z}$, $M_{2}^{z}$, and $M_{3}^{z}$ refer to the three bending moments measured at location 1,2, and 3 of the oar respectively. T and E are the beginning and the end of the blade respectively. ${\vec{F}}_{w,o}$ is the external force vector applied at the blade, while $F_{w,o}^{y^{\prime }}$ and $F_{w,o}^{x^{\prime }}$ are the perpendicular and parallel force components, respectively. ${\text{hl}\Delta \vec{oar}}_{1}$, ${\Delta \vec{oar}}_{2}$, ${\Delta \vec{oar}}_{T}$, and ${\Delta \vec{oar}}_{E}$ are the position vectors of location 1,2, T, and E in the loaded situation relative to their location in the unloaded position. Note that ${\Delta \vec{oar}}_{3}$ is not depicted in this figure since it is very small. The $r_{i}^{x^{\prime }}\text{s}$ and $r_{i}^{y^{\prime }}\text{s}$ represent the x and y-components of the known and measured moment-arms in a blade-bound frame of reference. $r_{PoA/T}^{x^{\prime }}$ is the x’-component of the position vector of the location of the PoA with respect to the beginning of the blade.

As outlined above, in theory, the presented method allows for an estimation of the unknown force vector and the position vector of the location of the PoA relative to a known location at the oar. To determine power loss due to the generation of propulsion according to Eq 1, these variables have to be combined with knowledge on the position and velocity of the oar in a world-bound frame of reference. However, in practice, the accuracy of the estimation of the force vector and its PoA remains to be shown. In contrast to our simple example discussed above, it is unrealistic to assume the oar shaft to be rigid [9]. This means that the pairs of strain gauges do not only have to provide information on bending moments, but also on the deflection of the oar. As of yet, the linearity of the relation between deflection of the oar and the local strain measured at location *i* of the tool is unknown. Moreover, in rowing the propulsion force consists of an unknown perpendicular and parallel force component relative to the orientation of the blade ($F_{w,o}^{y^{\prime }}$ and $F_{w,o}^{x^{\prime }}$ respectively; see Fig 2C in the material and methods section). As $F_{w,o}^{y^{\prime }}$ and its moment-arms are much larger than $F_{w,o}^{x^{\prime }}$ and its moment-arms, cross-talk may interfere with the determination of the parallel component. For sure, the parallel force cannot be determined if the oar is not bending, since it will result in zero lever-arms of $F_{w,o}^{x^{\prime }}$.

In an experimental study we therefore aim to evaluate whether the presented method can be used to obtain an accurate quantification of the net propulsion force vector applied at the blade of a rowing oar and the location of its PoA. More specifically, we will first confirm that strain gauges attached at different locations of the rowing oar allow for an accurate determination of $M_{i}^{z}\text{s}$ and the deflection of the oar. Additionally, we will examine whether the method provides an accurate quantification of both the perpendicular and parallel component of ${\vec{F}}_{w,o}$ and ${\vec{r}}_{PoA/w}$. Subsequently, we will examine the extent in which the power output associated with the bending of the oar can be determined accurately.

## Materials and methods

### Setup and instrumentation

A horizontal-plane experiment was conducted in a laboratory. One sweep oar (Big Blade; Concept2 Inc, Morrisville, USA) was instrumented with three pairs of strain gauges (HBM 1-DY41-6/350) measuring the local strain (2000 Hz) at three locations of the oar (see Fig 2 for the experimental setup and an overview of the frames of reference). The oar was supported at the oarlock and the end of the handle mimicking the oarlock and the rower’s hands. The part of the oar between the supports was assumed to be rigid, which means that the oar can be approached as a cantilever beam with one load applied at the free end. As can bee seen from Fig 2A, the net propulsion force vector (${\vec{F}}_{w,o}$) was simulated by pulling with varying force on a rope attached to the blade at different locations mimicking the ${\vec{r}}_{PoA/w}$ of the ${\vec{F}}_{w,o}$. The resultant force was measured using a force transducer (Futek LSB350, 500lbs, Futek, Irvine, USA; sample frequency of 2000 Hz). The direction of the force vector and the deflection of the oar were determined using 20 opto-electronic markers (Optotrak 3020, NDI, Ontario, Canada; sample frequency of 100 Hz) mounted at the oar, the oar blade, and the force transducer (see Fig 2 for the exact locations of the markers). Data obtained with Optotrak and the force transducer were considered to be the most accurate and used for (1) calibration of the output of the strain gauges, and (2) validation of the output variables of the presented method (see below). All sensor signals were recorded using two bridge modules (NL-9237, National Instruments, Austin,USA). In order to synchronize the signals an additional analog input channel was used to measure the start signal of the Optotrak system.

The experiment consisted of 12 trials in which time-varying forces (ranging between 0 and 400 N; based on estimated forces in on-water rowing studies [4]) were quasi randomly applied at the four different positions located at 0.225, 0.327, 0.423, and 0.520 m from the beginning of the blade. The angle of the resultant force ranged between 0 and 2.6 rad relative to the x-axis of the earth bound frame of reference (see Fig 2). Trials with an even number were used to calibrate the output of the strain gauges (from now on referred to as ‘calibration trials’), while trials with an uneven number (from now on called ‘validation trials’) were used to validate the obtained ${\vec{F}}_{w,o}$, ${\vec{r}}_{PoA/w}$, and the instantaneous power output associated with the deflection of the oar (*P*_*defl*_; see below).

### Calibration of strain gauges

In order to calculate ${\vec{F}}_{w,o}$, ${\vec{r}}_{PoA/w}$, and *P*_*defl*_, output of the strain gauges first had to be calibrated for (1) internal bending moments, (2) the deflection of the oar relative to its neutral position and (3) the orientation of the blade relative to an earth bound frame of reference ($M_{sg}^{z}$_*i*_, $\Delta_{oar_{sg}}^{y}$_*i*_, and $\Phi_{b/w_{sg}}$; see Fig 2). Note that the calculated internal bending moments only have a z-component since the analyses were restricted to forces and motions in the horizontal plane. Furthermore, deflection of the oar was only determined in y-direction, assuming deflection of the oar in x-direction to be negligible small. The deflection of the oar was calibrated for five locations at the oar: i.e. the locations where the strain gauges were attached, and the beginning and end of the blade (point T and E, respectively).

Using the data from the calibration trials, a linear relation was fitted between the output signals of every pair of strain gauges attached at location *i* and the related applied internal bending moments at location *i*. As the deflection of the oar at locations *i*, T, and E depend on the deflection of the previous locations, data of all strain gauges were used as inputs to calibrate the deflection of the oar. A similar method was used to calibrate Φ_*b*/*w*_.

### Determination of $F_{w,o}^{y^{\prime }}$, $F_{w,o}^{x^{\prime }}$, $r_{PoA/w}^{x^{\prime }}$, and *P*_*defl*_

#### Determination of estimated values

Assuming (1) the blade to be rigid under all circumstances, (2) the product of the inertia and oar angular acceleration to be negligible small, and (3) the x’- components of the moment-arms in a blade-bound frame of reference to be identical to the x-components of the moment-arms in an earth bound frame of reference, $F_{w,o_{sg}}^{x^{\prime }}$, $F_{w,o_{sg}}^{y^{\prime }}$, and $r_{PoA/w_{sg}}^{x^{\prime }}$ could be calculated using the approach outlined in the introduction. In this case, a closed system with three unknown parameters and three independent equations was constructed:

$$\begin{array}{c} M_{1}=\left(r_{T/1}^{x^{\prime }}+r_{PoA/T}^{x^{\prime }}\right)F^{y^{\prime }}-r_{T/1}^{y^{\prime }}F^{x^{\prime }} \end{array}$$ (7a)

$$\begin{array}{c} M_{2}=\left(r_{2/1}^{x^{\prime }}+r_{T/1}^{x^{\prime }}+r_{PoA/T}^{x^{\prime }}\right)F^{y^{\prime }}-r_{T/2}^{y^{\prime }}F^{x^{\prime }} \end{array}$$ (7b)

$$\begin{array}{c} M_{3}=\left(r_{2/3}^{x^{\prime }}+r_{1/2}^{x^{\prime }}+r_{T/1}^{x^{\prime }}+r_{PoA/T}^{x^{\prime }}\right)F^{y^{\prime }}-r_{T/3}^{y^{\prime }}F^{x^{\prime }} \end{array}$$ (7c)

In which $r_{i}^{x^{\prime }}\text{s}$ are assumed to be equal to the associated $r_{i}^{x}\;\text{s}$ and $r_{i}^{y^{\prime }}\text{s}$ are calculated as:

$$\begin{array}{c} r_{i}^{y^{\prime }}=\Delta_{oar_{sg}}^{y}\cdot \text{cos}\left(\Phi_{b/w_{sg}}\right)-r_{i}^{x}\cdot \text{sin}\left(\Phi_{b/w_{sg}}\right) \end{array}$$ (8)

$r_{PoA/w_{sg}}^{x^{\prime }}$ is calculated as the sum of $r_{T/w_{sg}}^{x^{\prime }}$ and $r_{PoA/T_{sg}}^{x^{\prime }}$.

The associated instantaneous power ($P_{defl_{sg}}$) was calculated as the dot product of ${\vec{F}}_{w,o_{sg}}$ and ${\dot{\vec{r}}}_{PoA/w_{sg}}$. To determine ${\dot{\vec{r}}}_{PoA/w_{sg}}$, ${\vec{r}}_{PoA/w_{sg}}$ was differentiated and rotated to the blade orientated frame of reference (see EQ A and B in S1 Appendix for an elaboration).

#### Determination of reference values

Reference values for ${\vec{F}}_{w,o\text{sg}}$ and ${\dot{\vec{r}}}_{PoA/w_{sg}}$, (i.e. $F_{w,o_{ref}}^{x^{\prime }}$, $F_{w,o_{ref}}^{y^{\prime }}$, and ${\dot{\vec{r}}}_{PoA/w_{ref}}$, respectively) were calculated using Optotrak and force transducer data. The reference value of $r_{PoA/w_{sg}}^{x^{\prime }}$ (i.e. $r_{PoA/w_{ref}}^{x^{\prime }}$) was obtained using a ruler. Reference power output values (i.e. $P_{defl_{ref}}$) were calculated as the dot product of ${\vec{F}}_{w,o_{ref}}$ and ${\dot{\vec{r}}}_{PoA/w_{ref}}$.

### Data analyses

Data analyses were performed using Matlab 2017A (the Mathworks Inc, Matick, Massachusetts, United States). Data collected with the strain gauges and force transducer, both measured with 2000 Hz, were downsampled to 100 Hz in order to match the sample frequency of the Optotrak.

Nine percent of the Optotrak data was missing. Cases with missing Optotrak data were excluded for further analysis. Additionally, cases in which the applied parallel force was lower than -30 N and higher than 20 N were excluded for further analyses since these values were considered to be unrealistic for rowing practice (based on findings of [4]). These exclusions resulted in a data set of 6 calibration trials consisting of 22445 samples and 6 validation trials consisting of 18432 samples.

### Statistical validation of the obtained results

Statistical analyses were performed using Matlab 2017A (the Mathworks Inc, Matick, Massachusetts, United States). First the validity of the obtained gains for $M_{sg}^{z}$_*i*_, $\Delta_{oar_{sg}}^{y}$_*i*_, and $\Phi_{b/w_{sg}}$ was checked. Subsequently, the correspondence between $F_{w,o_{sg}}^{y^{\prime }}$, $F_{w,o_{sg}}^{x^{\prime }}$, $r_{PoA/w_{sg}}^{x^{\prime }}$ and their related reference values was quantified using intraclass correlation coefficients (ICC(3.1)) [12], since this reflects deviation from the identity line. ICC values between.75 and.90 were interpreted as reasonably good, while ICC values higher than.90 were assumed to be good (based on [13] in [12]). In addition, the standard error of the estimate (SEE) was calculated to provide dispersion of the prediction. $P_{defl_{sg}}$ and $P_{defl_{ref}}$ were compared to provide an indication of the maximum accuracy with which $P_{defl_{sg}}$ may be estimated during on-water rowing.

## Results

### Typical examples

**Fig 3:**
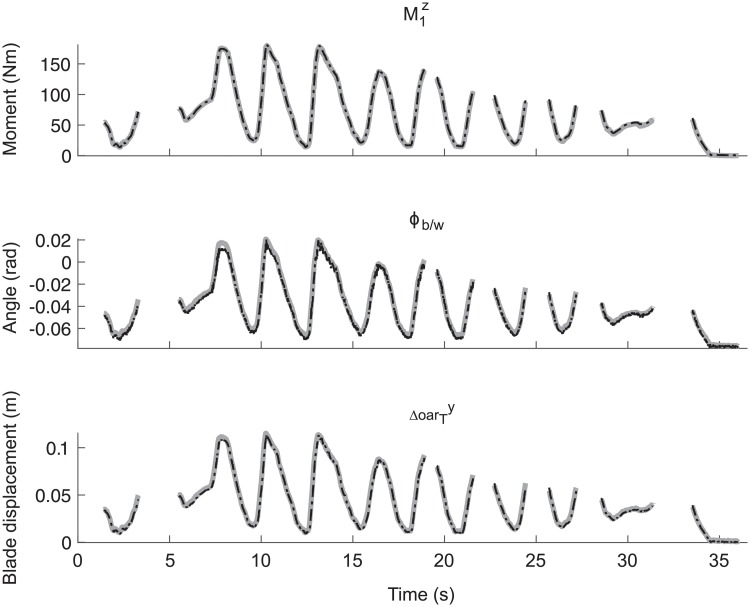
Typical examples of the (1) bending moment at one location of the oar $M_{1}^{z}$, (2) the orientation of the blade relative to an earth-bound frame of reference (Φ_*b*/*w*_), and (3) the displacement of the beginning of the blade in y-direction for one validation trial ($\Delta_{oar_{T}}^{y}$). Reference values are depicted using a bold grey line, while the values estimated using strain gauges are illustrated as dashed black lines. Note that the missing data refers to data in which the parallel force is lower than -30 N or higher than 20 N.

In Fig 3 typical examples of an estimated bending moment and the orientation of the blade plus their references are shown for one validation trial in order to provide an indication of the accuracy of the estimated values. Likewise, the estimated displacement of the beginning of the blade in y-direction and its reference are depicted. These examples imply that output of the strain gauges can be calibrated for bending moments, the deflection of the oar, and the orientation of the blade relative to the earth-bound frame of reference (see Table A in S1 Table for correspondence values).

**Fig 4:**
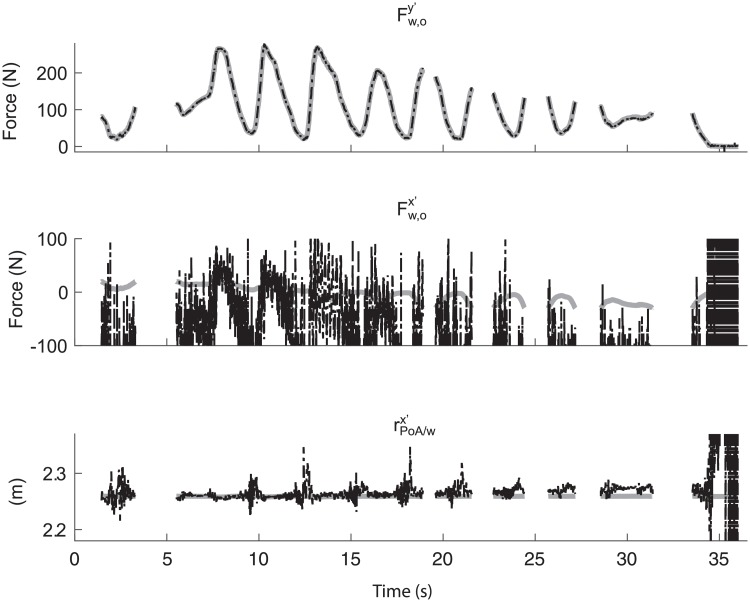
Typical examples of (1) the perpendicular force component ($F_{w,o}^{y^{\prime }}$), (2) the parallel force component ($F_{w,o}^{x^{\prime }}$), and (3) the x’-component of the location of the point of application $r_{PoA/w_{sg}}^{x^{\prime }}$. The bold grey lines represent the reference values obtained using Optotrak and the force transducer, while the black dashed lines are the values obtained using the presented method. Note that the missing data is data in which the parallel force is lower than -30 N or higher than 20 N.

In Fig 4, typical examples of the estimated $F_{w,o_{sg}}^{y^{\prime }}$, $F_{w,o_{sg}}^{x^{\prime }}$, and $r_{PoA/w_{sg}}^{x^{\prime }}$ and their references are presented for the same validation trial. These typical examples show that $F_{w,o_{sg}}^{y^{\prime }}$ is very similar to $F_{w,o_{ref}}^{y^{\prime }}$, while $F_{w,o_{sg}}^{x^{\prime }}$ is very different from $F_{w,o_{ref}}^{x^{\prime }}$. $r_{PoA/w_{sg}}^{x^{\prime }}$ seems to be fairly similar to $r_{PoA/w_{ref}}^{x^{\prime }}$ when there is a force applied at the oar.

### Accuracy of $\Phi_{b/w_{sg}}$, ${\vec{F}}_{w,o\text{sg}}$, and $r_{PoA/w_{sg}}^{x^{\prime }}$

Overall, correspondence values between $F_{w,o_{sg}}^{y^{\prime }}$ and $F_{w,o_{ref}}^{y^{\prime }}$ were very good, while there was no agreement between $F_{w,o_{sg}}^{x^{\prime }}$ and $F_{w,o_{ref}}^{x^{\prime }}$, and $r_{PoA/w_{sg}}^{x^{\prime }}$ and $r_{PoA/w_{ref}}^{x^{\prime }}$ (see Table 2 for all correspondence values).

**Table 2:** Correspondence values (i.e. Intra Class Correlation; ICC; and the Standard Error of the Estimate; SEE) between estimated force components and the x’-component of the position vector of the location of the point of application (i.e. $F_{w,o_{sg}}^{y^{\prime }}$, $F_{w,o_{sg}}^{x^{\prime }}$,$r_{PoA/w_{sg}}^{x^{\prime }}$, respectively) on the one hand, and their reference values on the other hand for the (1) whole data set and a data set that only includes samples of which the displacement of the beginning of the blade was more than (2) 0.58 cm and (3) 2.6 cm.

	ICC	SEE
$F_{w,o_{sg}}^{y^{\prime }}$		
all data	.999	3.8 N
$\Delta_{oar_{ref_{P}}}^{y}\geq .0058\;\text{m}$	.999	4.0 N
$\Delta_{oar_{ref_{P}}}^{y}\geq .262\;\text{m}$	.998	4.6 N
$F_{w,o_{sg}}^{x^{\prime }}$		
all data	.000	67503 N
$\Delta_{oar_{ref_{P}}}^{y}\geq .0058\;\text{m}$	.021	279.2 N
$\Delta_{oar_{ref_{P}}}^{y}\geq .262\;\text{m}$	.238	83.7 N
$r_{PoA/w_{sg}}^{x^{\prime }}$		
all data	.000	15.29 m
$\Delta_{oar_{ref_{P}}}^{y}\geq .0058\;\text{m}$	.927	.047 m
$\Delta_{oar_{ref_{P}}}^{y}\geq .262\;\text{m}$	.992	.015 m

However, a detailed exploration of the data revealed that correspondence values between $r_{PoA/w_{sg}}^{x^{\prime }}$ and $r_{PoA/w_{ref}}^{x^{\prime }}$ were related to the deflection of the oar. As can be seen in Table 2 correspondence between $r_{PoA/w_{sg}}^{x^{\prime }}$ and $r_{PoA/w_{ref}}^{x^{\prime }}$ was good (ICC≥.900) when the beginning of the blade was displaced with more than 0.58 cm, which was related to a perpendicular force of 6.0 N. SEE was still relatively high but decreased when the oar was bending more. SEE was smaller than 1.5 cm when the displacement of the beginning of the blade was more than 2.6 cm, which corresponds with a perpendicular force of higher than 42.6 N.

### Accuracy of $P_{defl_{sg}}$

**Fig 5:**
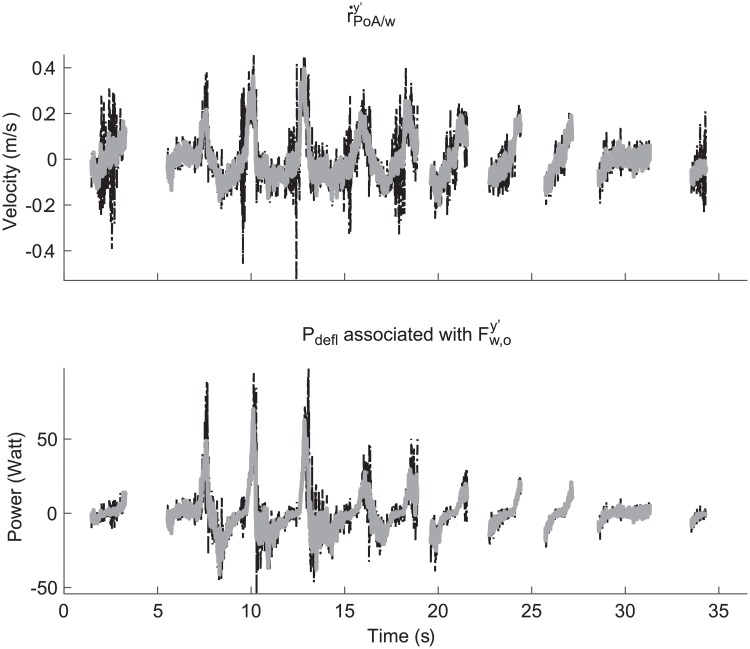
Typical examples of (a) the velocity in y’-direction of the location of the blade where the point of application is located (${\dot{r}}_{PoA/w_{sg}}^{y^{\prime }}$), and (b) the component of *P*_*defl*_ associated with the perpendicular force. The bold grey lines represent the reference values obtained using Optotrak and the force transducer, while the black dashed lines are the values obtained using the presented method. Note that the missing data is data in which the parallel force is lower than -30 N and higher than 20 N.

As $F_{w,o_{sg}}^{x^{\prime }}$ could not be determined accurately, instantaneous power output associated with $F_{w,o_{sg}}^{x^{\prime }}$ could not be determined. Correspondence between the estimated instantaneous power output associated with $F_{w,o_{sg}}^{y^{\prime }}$ using the strain gauges and its reference value was reasonably accurate (ICC = .747, SEE = 14.15; see Fig 5).

## Discussion

In this article we presented a method in which we used the bending oar moments measured with strain gauges to determine the net propulsion force vector and its ${\vec{r}}_{PoA/w}$ in rowing. Additionally, we validated the accuracy of the obtained force vector and its ${\vec{r}}_{PoA/w}$ for a simulated rowing situation. We confirmed that output of the strain gauges attached at a rowing oar shaft can be accurately calibrated for (1) internal bending moments, (2), the deflection of the oar, and (3) the orientation of the blade relative to an earth-bound frame of reference. Most importantly, we found that the perpendicular component of the propulsion force vector ($F_{w,o}^{y^{\prime }}$) could be validly obtained. Moreover, we found that ${\vec{r}}_{PoA/w}$ could be accurately determined when the beginning of the blade was displaced with more than.58 cm in y-direction, which corresponds to a perpendicular force of 6.0 N for this particular oar. Additionally, we found that an increase in the perpendicular force, resulted in a more accurate determination of ${\vec{r}}_{PoA/w}$. Subsequently, we have shown that the power output associated with the perpendicular force resulting in bending of the oar could be determined with reasonable accuracy. The parallel force component could not be estimated.

Using a different measuring setup, Hofmijster and colleagues [4], were -in contrast to us- able to estimate the parallel net water force component. They cut the oar and mounted a custom-built oar shaft with two strain gauges each in an angle of 45 degrees relative to the length of the oar. This custom-made oar shaft was designed to be sensitive for strain caused by the parallel force, but added considerable mass to the oar. Moreover, this was a one-off setup. In the context of a light-weight and practical method, we measured local strain by using pairs of strain gauges that were mounted directly at the oar shaft itself. In the current study, the strain of the oar shaft caused by the parallel force might have been too small to be distinguished from noise. Additionally, cross-talk due to strain caused by the perpendicular force may have interfered with the determination of the parallel force as well. In pilot studies we have aimed to obtain the parallel force by measuring the compression and extension of the oar with strain gauges. However, the parallel forces could still not be obtained using that method due to the combination of high stiffness of the shaft and low parallel forces resulting in very small deformations of the oar shaft in x’-direction. As the parallel force does result in additional power loss [4], future studies should keep on searching for a practical method that allows for an accurate estimation of the parallel force component.

However, the presented method is—to our knowledge—the first method that allows for a better estimation of the time-dependent variation in ${\vec{r}}_{PoA/w}$ in on-water rowing as opposed to previous studies in which it was commonly assumed that the PoA is fixed in the center of the blade [1–8]. This improvement in determination is expected to result in a more accurate determination of power loss at the blade during on-water rowing relative to previous estimations as (1) the ${\vec{r}}_{PoA/w}$ fluctuates during the stroke [10], and (2) the actual ${\vec{r}}_{PoA/w}$ highly influences calculated values of power loss at the blade [4].

Some limitations related to the setup or the experiment are worth mentioning. Firstly, trials with time-varying forces have been used to calibrate the strain gauges. These forces were applied manually by pulling a rope that was attached to the blade of the oar. In hindsight, a static controlled calibration might have been preferred for calibration, since gains for linear fits also depend on the distribution of the input variables. For example, since the forces were manually applied during the trials relatively many samples reflect a bending moment of 0 Nm and only a few samples are related to a max bending moment. This may have influenced the gains for calibration. However, sensitivity tests in which the distribution of the input variables has been equalized did not reveal different agreement values for the estimated water force vector and the ${\vec{r}}_{PoA/w}$. Secondly, in this experiment the point of application was fixed at the blade during the trials, while this is assumed to vary during the stroke in on-water rowing. However, as the same system of equations will hold true for a time-varying point of application, we do not have doubts about the generalization of our results with respect to the determination of the location of the point of application.

With respect to the generalization of our results to rowing practice a few concerns are worth mentioning. Firstly, we evaluated the presented method for one type of oar with specific stiffness properties. Although the same system of equations holds true for different rowing oars, force thresholds and maybe even displacement thresholds for which the method provides valid insight in ${\vec{r}}_{PoA/w}$ may differ. A calibration and quick validation of the presented method for different oars is thus highly recommended. Secondly, it should be noted that the current calculated power output values associated with the perpendicular force are expected to be much smaller than the power output values associated with the perpendicular water force component in real on-water rowing, as the perpendicular velocity of the location at the blade at which the ${\vec{F}}_{w,o}$ is applied (${\dot{r}}_{PoA/w}^{y^{\prime }}$) will be larger in on-water rowing. In this experiment, ${\dot{r}}_{PoA/w}^{y^{\prime }}$ was only due to the bending of the oar, while in on-water rowing ${\dot{r}}_{PoA/w}^{y^{\prime }}$ consists of three components that are all different from zero: (1) a velocity component that is due to the velocity of the boat, (2) a velocity component that is due to the rotation of the (rigid) oar relative to the boat, and (3) a velocity component that is due to the bending of the oar (see also the appendix on the calculations of ${\dot{r}}_{PoA/w}^{y^{\prime }}$). To determine the total instantaneous power loss due to the generation of propulsion in on-water rowing and thus ${\bar{P}}_{blade}$, the velocity components related to the boat velocity and the oar angular velocity need to be taken into account as well.

This study mainly focused on rowing. In passing, we note that the essence of the presented method—using strain gauges to measure bending moments and a system of equations to determine the unknown parameters related to external forces and the position of the PoA—may well be suited to be used for accurate quantifications of force vector components and the associated position of the PoA in other (sport) applications, such as kayaking and different ball sports. For example, the application of the presented method may be interesting for obtaining (bio)mechanical information in ball sports where athletes hit a ball with a racket or bat.

## Conclusion and relevance

The aim of this study was to describe and evaluate a method that allows for an accurate determination of the power loss due to the generation of propulsion in rowing. As mentioned in the introduction, an accurate quantification of the water force vector, the ${\vec{r}}_{PoA/w}$, and its time-derivative are crucial for obtaining insight in that power loss. Despite the fact that the parallel force component relative to the blade could not be obtained, we are the first who developed a cost-effective practical method that allows for the determination of a perpendicular force component in combination with its time-varying ${\vec{r}}_{PoA/w}$ in on-water rowing practice. The presented method is therefore a promising option to gain more insight in the power losses due to the generation of propulsion during on-water rowing.

## Supporting information

S1 Appendix Determination of the time-derivative of the point of the blade where the water force vector is applied. (PDF)

S1 Table Correspondence values between the estimated bending moments, the displacement of the oar, and the angle of the blade relative to its neutral position on the one hand and their reference values on the other hand. (PDF)
