# Supplementary material for: Towards determination of power loss at a rowing blade: Validation of a new method to estimate blade force characteristics
Source: PLoS One. 2019 May 9;14(5):e0215674. doi: 10.1371/journal.pone.0215674 (PMC6508922; doi:10.1371/journal.pone.0215674)
Supplement: S1 Appendix — (PDF) [file pone.0215674.s001.pdf]

S1 Appendix

Determination of the time derivative of the point of the blade where the water force vector is applied ($\dot{\vec{r}}_{PoA/w_{sg}}$). The expression of $\vec{r}_{PoA_{sg}}$ in an earth bound frame of reference is:

$$\begin{aligned}\vec{r}_{PoA/w_{sg}}(t) =& \vec{r}_{d/w} + |\vec{r}_{T1/d}| \cdot \begin{pmatrix}\cos(\Phi_{d/w})\\ \sin(\Phi_{d/w})\end{pmatrix}\\ &+ \Delta^{y}_{oar_{sg_T}} \cdot \begin{pmatrix}-\sin(\Phi_{d/w})\\ \cos(\Phi_{d/w})\end{pmatrix}\\ &+ |\vec{r}_{PoA/T_{sg}}| \cdot \begin{pmatrix}\cos(\Phi_{d/w} + \Phi_{b/d_{sg}})\\ \sin(\Phi_{d/w} + \Phi_{b/d_{sg}})\end{pmatrix}\end{aligned} \tag{A}$$

where $\vec{r}_{d/w}$ is the position vector of the oar pin in an earth-bound frame of reference. While in real rowing this information may be provided by a GPS, in our experiment the origin of the frames of references is at the oar pin and the $\vec{r}_{d/w}$ is thus zero. $\vec{r}_{T1/d}$ is the position vector of the beginning of the blade in the unloaded position. Calculation of $\vec{r}_{T/d}$ is based on the angle of the oar pin relative to the earth-bound frame of reference ($\Phi_{d/w}$) and the distance of the beginning of the blade from the oar. $\Delta^{y}_{oar_{sg_T}}$ is the position of the beginning of the blade in the loaded situation relative to the position of the beginning of the blade in the unloaded situation. Calculation of $\vec{r}_{PoA/T_{sg}}$ is based on $|r_{PoA/T_{sg}}|$ and the angle of the blade relative to an earth-bound frame of reference ($\Phi_{b/w_{sg}} = \Phi_{d/w} + \Phi_{b/d_{sg}}$), both determined using the presented method.

Therefore, $\dot{\vec{r}}_{PoA/w_{sg}}$ is:

$$
\begin{aligned}
\dot{\vec{r}}_{PoA/w_{sg}} =& \dot{\vec{r}}_{d/w} + |\vec{r}_{T/d}| \cdot \dot{\Phi}_{d/w} \cdot \begin{pmatrix} -\sin(\Phi_{d/w}) \\ \cos(\Phi_{d/w}) \end{pmatrix} \\
&+ \Delta \dot{oar}^{y}_{sg_T} \cdot \begin{pmatrix} -\sin(\Phi_{d/w}) \\ \cos(\Phi_{d/w}) \end{pmatrix} + \Delta oar^{y}_{sg_T} \cdot \dot{\Phi}_{d/w} \cdot \begin{pmatrix} -\cos(\Phi_{d/w}) \\ -\sin(\Phi_{d/w}) \end{pmatrix} \\
&+ |\dot{r}_{PoA/T_{sg}}| \cdot \begin{pmatrix} \cos(\Phi_{d/w} + \Phi_{b/d_{sg}}) \\ \sin(\Phi_{d/w} + \Phi_{b/d_{sg}}) \end{pmatrix} \\
&+ |r_{PoA/T_{sg}}| \cdot (\dot{\Phi}_{d/w} + \dot{\Phi}_{b/d_{sg}}) \cdot \begin{pmatrix} -\sin(\Phi_{d/w} + \Phi_{b/d_{sg}}) \\ \cos(\Phi_{d/w} + \Phi_{b/d_{sg}}) \end{pmatrix}
\end{aligned} \tag{B}
$$
