# Supplementary material for: Towards determination of power loss at a rowing blade: Validation of a new method to estimate blade force characteristics
Source: PLoS One. 2019 May 9;14(5):e0215674. doi: 10.1371/journal.pone.0215674 (PMC6508922; doi:10.1371/journal.pone.0215674)
Supplement: S1 Table — (PDF) [file pone.0215674.s002.pdf]

S1 Table

Table A. Correspondence values (i.e. Intra Class Correlation; ICC; and the Standard Error of the Estimate; SEE) between estimated bending moments, the displacement of the oar and angle of the blade relative to its neutral position ($M^z_{sg\,i}$, $\Delta^y_{oar_{sg}\,i}$ and $\Phi_{b/wsg}$) on the one hand and their reference values -indicated with subscript 'ref' in the main text- on the other hand.

	**ICC**	**SEE**
$M^z_{sg\,1}$	1.000	3.48 Nm
$M^z_{sg\,2}$	1.000	1.77 Nm
$M^z_{sg\,3}$	1.000	1.42 Nm
$\Delta^y_{oar_{sg}\,1}$	.991	.0003 m
$\Delta^y_{oar_{sg}\,2}$	.998	.0012 m
$\Delta^y_{oar_{sg}\,3}$	.998	.0015 m
$\Delta^y_{oar_{sg}\,P}$	.998	.0022 m
$\Delta^y_{oar_{sg}\,E}$	.998	.0034 m
$\Phi_{b/wsg}$	.998	.0025 rad
